# Extraction, Purification, Structural Characteristics, Biological Activities, and Applications of the Polysaccharides from *Zingiber officinale* Roscoe. (Ginger): A Review

**DOI:** 10.3390/molecules28093855

**Published:** 2023-05-02

**Authors:** Wenjing Hu, Aiqi Yu, Shuang Wang, Qianxiang Bai, Haipeng Tang, Bingyou Yang, Meng Wang, Haixue Kuang

**Affiliations:** Key Laboratory of Basic and Application Research of Beiyao, Ministry of Education, Heilongjiang University of Chinese Medicine, Harbin 150040, China

**Keywords:** *Zingiber officinale* Roscoe, polysaccharide, structural characteristic, biological activity, structure–activity relationship, application

## Abstract

*Zingiber officinale* Roscoe. (ginger) is a widely distributed plant with a long history of cultivation and consumption. Ginger can be used as a spice, condiment, food, nutrition, and as an herb. Significantly, the polysaccharides extracted from ginger show surprising and satisfactory biological activity, which explains the various benefits of ginger on human health, including anti-influenza, anti-colitis, anti-tussive, anti-oxidant, anti-tumor effects. Here, we systematically review the major studies on the extraction and purification of polysaccharides from ginger in recent years, the characterization of their chemical structure, biological activity, and structure–activity relationships, and the applications of ginger polysaccharides in different fields. This article will update and deepen the understanding of ginger polysaccharide and provide a theoretical basis for its further research and application in human health and product development.

## 1. Introduction

Ginger is a perennial herb of the Zingiberaceae family, scientifically named *Zingiber officinale* Roscoe. [1,2]. As early as the 18th century, Europeans began using ginger to make beer, candy, bread, biscuits, and so on. It is also an indispensable seasoning in Japanese cuisine, Korean food, and Chinese food [3,4]. Therefore, it is a widely distributed plant with a long cultivation history. It is now mainly cultivated and used in India, Nigeria, China, Burma, Indonesia, Australia, Japan, Sri Lanka, Germany, Greece, Arabia, and other countries. Among them, India, Nigeria, and China are the main producers of ginger, according to the Food and Agriculture Organization of the United Nations statistical database (FAO) in 2022 [5,6]. As an important plant with economic, ornamental, and even edible and medicinal values, it has attracted attention from multiple scientific fields. It is a rich source of nutrients such as protein, vitamins, minerals, fat, and crude fiber [7,8,9]. It has attracted increasing interest from nutrition researchers and health-conscious consumers.

**Figure 1:**
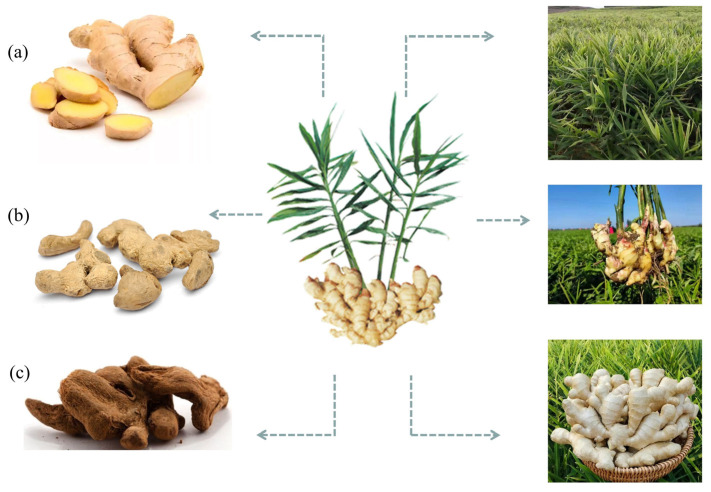
A plant image of *Zingiber officinale* Roscoe. (ginger). (**a**) Shengjiang (fresh ginger); (**b**) Ganjiang (dried ginger); (**c**) Paojiang (fried ginger).

In its long history of application and consumption, ginger has been fully utilized and deeply developed as a nourishing food and traditional oriental medicine [10,11,12]. As a “medicinal food homology” plant, ginger and its active ingredients are not only used as delicious food, but also as effective empirical medicine [13,14]. Ginger can be used as tea or health drinks and is traditionally used to treat diseases such as colds, vomiting, and fatigue [15,16,17]. In Africa, ginger essential oil is commonly used to relax muscles or treat muscle and joint pain, swelling, and inflammation. It can also be dripped in water and gargled to relieve toothache. In addition, ginger is an important part of Ayurveda preparation “Trikatu”. Trikatu can be used in combination with other drugs to treat asthma, bronchitis, dysentery, fever, and intestinal infections [18]. Due to its health benefits such as enhancing immunity and promoting energy metabolism, ginger is widely used as a restorative supplement or medicinal food in folk [19,20]. Officially, the Chinese Pharmacopoeia 2020 version includes three related products, including Shengjiang (fresh ginger), Ganjiang (dried ginger), and Paojiang (fried ginger) (Figure 1). Therefore, the nutritional and medicinal value of ginger has been widely recognized, meeting the needs of consumers for a healthy diet, nutritional intake, and dietary treatment.

Over the past few decades, many studies have been conducted on ginger around the world. It is widely believed that ginger can be used as a nutritious vegetable or natural functional food. As ginger contains important phytochemicals and biologically active ingredients, such as volatile oil, curcumin, flavonoids, and polysaccharides, it is increasingly popular in daily diet [21,22,23,24]. Gingerol, as an important source of ginger’s pungent taste, endows ginger with a unique spicy taste and is also one of the main active ingredients in ginger, which is often the focus of researchers [25,26,27]. However, the macromolecular compound polysaccharides obtained from ginger exhibit surprising and satisfactory biological activities, which may explain their various benefits to human health, including anti-influenza, anti-colitis, anti-tussive, anti-oxidant, and anti-tumor effects [28,29]. Currently, various polysaccharides have been extracted from ginger through different extraction and purification methods. Due to the diversity of the chemical structure of polysaccharides in ginger, as well as their different physicochemical properties and biological activities, it has attracted more and more research interest. Ginger polysaccharides are considered safe and non-toxic. At the same time, they have a variety of beneficial functions for the body, and have a good development prospect in food, cosmetics, medicine, and other industries [30,31].

Reviewing the existing and available literature, although there are some review articles on ginger and its bioactive components, they mainly focus on small molecular components such as flavonoids, polyphenols, and alkaloids; there is a lack of comprehensive reviews focusing solely on the macromolecular polysaccharides of ginger. At the same time, the research on ginger polysaccharide is increasing gradually, becoming more complex and sophisticated. To avoid duplication and confusion of research work, there is an urgent need to sort out the existing research on ginger polysaccharides. Therefore, this paper systematically reviews recent major studies on ginger polysaccharide-related extraction and purification methods and characterizes their chemical structures, biological activity, and structure–activity relationship, and the applications of ginger polysaccharides in different fields to provide reference for further research on ginger polysaccharides and the development of related functional products.

## 2. Extraction and Purification Methods of Ginger Polysaccharides

### 2.1. Extraction Methods of Ginger Polysaccharides

Effective extraction and purification of ginger polysaccharides is the main premise for studying the structures and biological activities of polysaccharides. In order to maximize the extraction efficiency of bioactive macromolecular polysaccharides from ginger, researchers have carried out a series of explorations into various extraction strategies, including the traditional extraction method of solvent extraction method (SEE), the novel extraction methods of enzyme-assisted extraction (EAE), and ultrasonic-assisted extraction (UAE). The specific information of various extraction methods is summarized in Table 1.

#### 2.1.1. Solvent Extraction Method

Among the different extraction methods, such as SEE, UAE, and EAE, SEE is the most commonly used method for extracting ginger polysaccharides because of its economical nature and convenient use. Extraction temperature varies from 50 °C to 100 °C, the extraction time usually ranges from 1 h to 5 h, and the total yield ranges from 6.83% to 12.13%. It is worth noting that in alkaline environment, ginger cells are more easily destroyed, and the extraction rate of polysaccharides is higher. The results showed that the yield of ginger polysaccharide extracted with distilled water was 6.83 ± 0.54%, while the yield of polysaccharide extracted with alkaline water was 11.38 ± 1.17% [32]. Although the traditional extraction method has certain advantages, it has significant disadvantages, such as long extraction time, high extraction temperature, low extraction efficiency, and high risk for degradation of polysaccharides [33,34]. Hence, the application of the extraction method is limited. Therefore, it is necessary to develop an economical and highly efficient method for the extraction of polysaccharides from ginger.

#### 2.1.2. Ultrasonic-Assisted Extraction Method

As a non-thermal physical processing technology, UAE has significant advantages in extracting ginger polysaccharides. The research shows that the extraction of ginger polysaccharides (GSLPs) by UAE takes 50 min, while the hot water extraction (HWE) takes 5 h. Compared with the HWE method, UAE technology can greatly reduce time consumption and improve the GSLP’s preparation efficiency. The total sugar content of GSLP extracted by UAE was 44.65 ± 0.95%, while that extracted by HWE was 37.85 ± 0.55%. This may be related to the fact that the cavitation effect of ultrasound is more effective in destroying the cell wall and cell membrane of plants and enhancing the contact between solvents and polysaccharides, thus promoting the dissolution and release of polysaccharides in cells [35,36]. The low total sugar content of HWE is due to the relatively high extraction temperature, which will also promote the dissolution of some non-polysaccharide components in the process of accelerating the dissolution and diffusion of polysaccharides into aqueous solution, thus reducing the total sugar content. Furthermore, the yield of GSLP extracted by HWE and UAE was 6.83 ± 0.54% and 8.29 ± 0.31%, respectively. It can be seen that UAE has a higher extraction rate than HWE. The yield of polysaccharides extracted from plant raw materials is largely affected by the cell wall and polysaccharide dissolution efficiency. HWE only slightly breaks the ginger cell wall, but the UAE method makes the structure of the cell wall and cell membrane of ginger stem and leaf more damaged and hydrolyzed, thus promoting the dissolution of polysaccharides. Therefore, from the perspective of extraction rate, the UAE method is a better choice to extract ginger stem and leaf polysaccharides [32].

Because the UAE of ginger polysaccharides has the advantages of high efficiency and low energy consumption, researchers have paid more attention to the UAE of polysaccharides in recent years [37]. Ultrasonic technology has developed to a great extent, and multi-frequency ultrasonic technology has gradually entered the vision of researchers. Multi-frequency UAE is based on the combination of multiple frequencies. Compared with traditional single-frequency ultrasound, multi-frequency ultrasound has low energy consumption, low noise, and obvious cavitation effect. It is reported that the efficiency of multi-frequency UAE of polysaccharides is higher than that of single-frequency ultrasound. It was found that the yield of polysaccharides extracted by tri-frequency ultrasound and dual-frequency ultrasound was 10.50 ± 0.20% and 9.74 ± 0.30%, respectively, higher than that extracted by single-frequency ultrasound (8.29 ± 0.31%) [32,38]. This may be due to the uniform energy distribution and easy resonance of extracted raw materials in dual-frequency and triple-frequency ultrasonic treatment, while single-frequency ultrasonic treatment has a directional effect. It can be seen that multi-frequency ultrasound can significantly improve the extraction rate of polysaccharides, which is more conducive to scientific research and industrial production.

#### 2.1.3. Enzyme-Assisted Extraction Method

Plant polysaccharides, one of the essential substances for maintaining normal life activities, are present in plant cell walls, as are ginger polysaccharides [39,40]. The suitable extraction method is to achieve high-efficiency extraction by enhancing the decomposition of cell walls without destroying the basic characteristics of ginger polysaccharides. EAE is the use of the enzyme specificity to destroy the plant cell wall under certain conditions, so that polysaccharides can be dissolved from the cell. The main influencing factor of this extraction method is enzymes. Enzymes react with accurate specificity and region selectivity and can maintain the biological activity of compounds [41,42]. EAE technology has been proved to have an edge over conventional techniques in sustainable polysaccharide extraction. The findings of Liao et al. (2020) showed that the extraction conditions of ginger polysaccharides obtained by EAE were 40 °C and 2 h, while the extraction conditions of ginger polysaccharides extracted by hot water were 100 °C and 4 h. Obviously, EAE has an advantage in economizing time [43]. In another study conducted by Chen et al., two polysaccharides (EGP1 and EGP2) were extracted from the ginger using the EAE method under certain reaction conditions. Compared with the hot water extraction method, enzyme-assisted extraction not only shortened the extraction time but also increased the yield of ginger polysaccharide from 6.83% to 8.13% [32]. Therefore, compared with the traditional hot water extraction method, this method has the advantages of short time, low energy consumption, good repeatability, and high extraction rate [44].

**Table 1:** A summary of ginger polysaccharides extraction methods.

Polysaccharide Fraction	Extraction Methods	Time (min)	Temperature (°C)	Solid–Liquid Ratio (g/mL)	Total Yield (%)	Ref.
Ginger polysaccharide (GPS)	Complex-enzyme hydrolysis extraction	60 min	55 °C	1:25	22.18%	[45]
Ginger pomace polysaccharides extracted by hot water (HW-GPPs)	Hot water extraction	120 min	70 °C	1:40	12.13 ± 1.15%	[46]
Ginger pomace polysaccharides extracted by ultrasonic–assisted (UA-GPPs)	Ultrasonic assisted extraction	17 min	74 °C	1:40	16.62 ± 1.82%	[46]
A neutral ginger polysaccharide fraction (NGP)	Hot water extraction	180 min	90 °C	1:20	N/A	[47]
A water extracted polysaccharides (WEP) containing fraction from ginger rhizome	Hot water extraction	60 min	100 °C	N/A	N/A	[48]
Crude ginger polysaccharides were extracted by hot water extraction (HCGP)	Hot water extraction	240 min	100 °C	1:20	11.74 ± 0.23%	[43]
Crude ginger polysaccharides were extracted by enzyme assisted extraction (ECGP)	Enzyme assisted extraction	120 min	40 °C	1:25	7.00 ± 0.04%	[43]
Crude ginger polysaccharides were extracted by ultrasonic cell grinder extraction (UCGP)	Ultrasonic cell grinder extraction	30 min	N/A	1:25	18.06 ± 0.05%	[43]
Polysaccharides from ginger (*Zingiber officinale* Roscoe.) stems and leaves (GSLP)	Hot water extraction	300 min	100 °C	1:20	6.83 ± 0.54%	[32]
Polysaccharides from ginger (*Zingiber officinale* Roscoe.) stems and leaves (GSLP)	Ultrasound-assisted extraction	60 min	50 °C	1:20	8.29 ± 0.31%	[32]
Polysaccharides from ginger (*Zingiber officinale* Roscoe.) stems and leaves (GSLP)	Alkaline solution extraction	120 min	25 °C	1:20	11.38 ± 1.17%	[32]
Polysaccharides from ginger (*Zingiber officinale* Roscoe.) stems and leaves (GSLP)	Enzyme-assisted extraction	90 min	50 °C	1:20	8.13 ± 0.85%	[32]
A novel polysaccharide (ZOP) was extracted from *Zingiber officinale* Roscoe.	Ultrasonic assisted extraction	120 min	90 °C	1:30	N/A	[49]
Ginger polysaccharide (GP)	Hot water extraction	60 min	100 °C	1: 20	N/A	[29]

N/A means not mentioned.

### 2.2. Purification Methods of Ginger Polysaccharides

**Figure 2:**
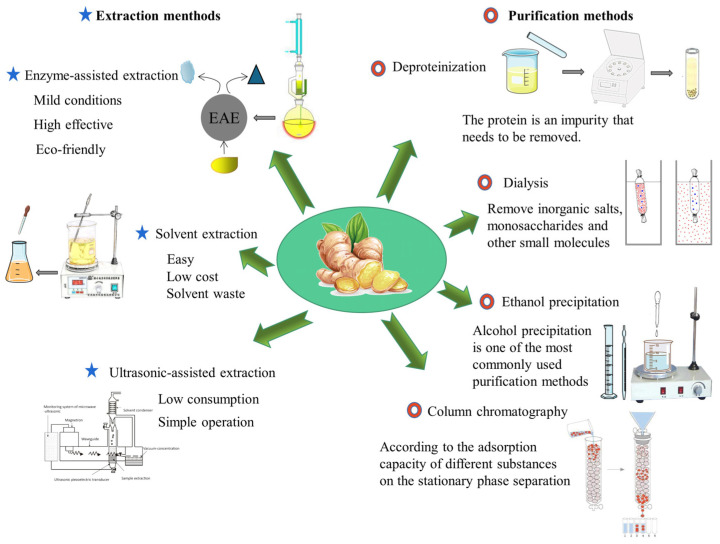
Methods for extraction and purification of ginger polysaccharides.

Ginger crude polysaccharideoften contains a variety of impurities, and it usually includes pigments, proteins, and inorganic salts. So, it needs to be purified further (Figure 2). Pigments can be removed by adsorption of macroporous resin and activated carbon. The commonly used deproteinization methods are the Sevag method, the trichloroacetic acid (TCA) method, and the enzymatic method [50,51]. Inorganic salts, monosaccharides, and other small molecular substances can be removed by dialysis and ultrafiltration. Next, the crude ginger polysaccharide solution needs to be further purified by column chromatography, and a suitable buffer solution is used as the mobile phase. Column chromatography is the most widely used method in the classification and purification of polysaccharides [52]. It can be divided into anion exchange chromatography and gel filtration chromatography. In the preparation of ginger polysaccharide GP1 and GP2, the crude ginger polysaccharide (GPS) was treated with S-8 macroporous resin column and the Sevag method to remove impurities, such as proteins, fragments, and nuclear acids, which can improve the purity and quality of GPS. Next, 0.1–1.0 mol/L NaCl solution was used as mobile phase, and DEAE-52 cellulose column was used for linear gradient elution. Subsequently, GP1 eluted with distilled water and GP2 eluted with 0.1 M NaCl buffer were further purified on Sephadex G-200 column. Finally, it was freeze dried [42]. In the preparation of neutral ginger polysaccharide (NGP), the crude ginger polysaccharide (CGP) was initially purified by DEAE-52 cellulose column, then further purified by Sephadex G-100 column, and finally freeze-dried to obtain purified NGP [47].

## 3. Structural Characteristics of Ginger Polysaccharides

The chemical structure of polysaccharides is the material basis of their biological activities. Ginger polysaccharides with different chemical structures have different pharmacological activities. Studying the structural characteristics of ginger polysaccharide is helpful to understand its pharmacological effects. Based on the extensive pharmacological effects of ginger polysaccharide, its structure research has been paid more and more attention. The structural characteristics of polysaccharides mainly include monosaccharide composition and sequence, molecular weight, positions of glycosidic linkages, and configuration. There are many analytical methods for the structure of polysaccharides, including not only instrumental analysis methods, such as high-performance liquid chromatography (HPLC), infrared spectroscopy (IR), nuclear magnetic resonance (NMR), gas chromatography (GC), mass spectrometry (MS), gas chromatography–mass spectrometry (GC-MS), etc., but also chemical methods, including methylation analysis, acid hydrolysis, periodate oxidation, and Smith degradation, as well as biological methods, such as specific glycosidase digestion, immunological methods, etc. Polysaccharides with various monosaccharide components and chemical structures have been isolated from ginger. The main structural characteristics of ginger polysaccharide, such as molecular weight and monosaccharide composition, are summarized in Table 2.

### 3.1. Molecular Weight

The most common molecular weight statistical values are number-average molecular weight (Mn), weight-average molecular weight (Mw), viscose-average molecular weight (Mη), and Z-average molecular weight (Mz). Generally, Mw and the weight fraction is used as the benchmark to measure the molecular weight. After the extraction and purification of ginger polysaccharide, many researchers have measured its Mw. Due to the different extraction and purification methods, the Mw of ginger polysaccharide is different. Ginger polysaccharides were extracted by HWE, EAE, and UCGE methods to obtain HGP, EGP1, EGP2, UGP1, and UGP2. Their relative molecular weights are 1831.75 kDa, 11.81 kDa, 688.73 kDa, 769.19 kDa, and 1432.80 kDa, respectively [43]. According to the existing literature, 26 kinds of polysaccharides were isolated from different parts of ginger, most of which were extracted from ginger rhizomes. The Mw of these polysaccharides ranges from 6.128 to 6040 kDa. Obtaining the size and distribution of molecular weight is important for understanding the structure of polysaccharides.

### 3.2. Monosaccharide Composition

Polysaccharides are polymers of monosaccharides linked by glycosidic bonds. Polysaccharides can be divided into homopolysaccharides and heteropolysaccharides according to whether they are composed of the same monosaccharide [53]. Many studies have shown that ginger polysaccharides are heteropolysaccharides and different studies have significant differences in the monosaccharide composition and molar ratio of polysaccharides extracted from ginger. However, most polysaccharides are composed of galactose (Gal), glucose (Glu), mannose (Man), arabinose (Ara), rhamnose (Rha), and xylose (Xyl). Interestingly, uronic acid, including glucuronic acid (GlcA), and galacturonic acid (GalA) have been detected in some polysaccharides. The diversity of monosaccharide composition and molar ratio in these polysaccharides is affected by many factors. For example, the monosaccharide composition of ginger polysaccharides HWE-GSLP extracted from ginger stems and leaves by the HWE method is Man:Rha:Glu:Gal:Xyl:Ara:GluA:GalA = 1.95:17.22:4.69:38.88:5.66:28.42:1.81:1.34. The monosaccharide composition of ginger polysaccharide GP extracted from ginger rhizome is Man:Rha:GlcA:GalA:Glc:Gal:Xyl:Ara:Fuc = 0.17:0.13:0.21:0.12:0.08:1:0.16:0.64:0.18. The monosaccharide composition of ginger polysaccharides extracted from ginger stems and leaves by EAE is Man:Rha:Glu:Gal:Xyl:Ara:GluA:GalA = 1.47:9.63:3.84:15.31:27.94:35.55:3.68:2.586 [29,32,46]. The difference in monosaccharide composition and molar ratio between these polysaccharides may be caused by the raw materials, extraction methods, separation, and purification methods, etc.

### 3.3. Chemical Structures

Polysaccharide has a more complex macromolecular structure than protein. The diversity of monosaccharides, connection methods, and complexity of branched chains make it difficult to identify their structures. Even so, over the years, researchers have extensively research on the chemical structure of ginger polysaccharides. Three different extraction methods (hot water extraction, enzyme assisted extraction, and ultrasonic cell grinder extraction) were used to separate crude polysaccharide fraction (GPs) from ginger, and they were separated into sub-fraction polysaccharides (HGP, EGP1, EGP2, UGP1, and UGP2) based on FT-IR and ^1^H and ^13^C NMR characterization. At the same time, these five polysaccharides contain the glycostatic linkage of →4)-α-d-Glc (1→ and -α-Manp-(1→, sharing similar spectra. In addition, EGP2 and UGP1 were also found to contain → 6)-β-d-Gal*p*-(1→, and UGP1 possessed more sulfate group [43]. In addition, the WEP fraction of ginger polysaccharide obtained by the HWE method contains branched chains α-glucan and a small amount of polygalacturonan. This glucan is connected by terminal-(1,4)-and (1,4,6)-α-Glc*p* residue composition. α-glucan was also found in another study [48]. A neutral heteropolysaccharide (NGP) was isolated from ginger and its physicochemical properties were analyzed. Through NMR and IR spectra, it is determined that NGP is α-glucan. The main chain of NGP is 1,4-linked α-d-Glc*p* and α-d-Glc*p* residues branched at C-6 position [47].

**Table 2:** Source, compound name, molecular weights, and monosaccharide composition of ginger polysaccharides.

Source	Compound Name	Molecular Weights	Monosaccharide Composition	Ref.
Rhizome	Ginger polysaccharide 2 (GP2)	12.619 kDa	Ara:Man:Glu:Gal = 4.78:16.70:61.77:16.75	[45]
Rhizome	Ginger polysaccharide 1 (GP1)	6.128 kDa	Man:Glu:Gal = 4.96:92.24:2.80	[45]
Rhizome	Ginger polysaccharide (GP)	N/A	Rha:Ara:Man:Glu:Gal = 3.64:5.37:3.04:61.03:26.91	[45]
Ginger pomace	Ginger pomace polysaccharide 1 extracted by hot water (HW-GPP1)	89.2 kDa	Man:Rha:Glu = 19.40 ± 0.06:12.27 ± 0.05:68.33 ± 0.24	[46]
Ginger pomace	Ginger pomace polysaccharide 2 extracted by hot water (HW-GPP2)	939.8 kDa	Man:Rha:Glu:Xyl:Ara = 11.84 ± 0.13:9.36 ± 0.02:58.05 ± 0.07:12.68 ± 0.15:8.07 ± 0.08.	[46]
Ginger pomace	Ginger pomace polysaccharide 3 extracted by hot water (HW-GPP3)	1007.9 kDa	Man:Rha:Glu:Gal:Xyl:Ara = 11.33 ± 0.05:13.90 ± 0.03:50.01 ± 0.13:10.96 ± 0.13:4.73 ± 0.09:9.07 ± 0.14	[46]
Ginger pomace	Ginger pomace polysaccharide 1 extracted by ultrasonic–assisted (UA-GPP1)	40.6 kDa	Man:Rha:Glu = 17.56 ± 0.11:7.72 ± 0.29:74.72 ± 0.27	[46]
Ginger pomace	Ginger pomace polysaccharide 2 extracted by ultrasonic–assisted (UA-GPP2)	868.1 kDa	Man:Rha:Glu:Xyl:Ara = 13.18 ± 0.05:9.03 ± 0.08:63.78 ± 0.14:8.97 ± 0.15:5.04 ± 0.08	[46]
Ginger pomace	Ginger pomace polysaccharide 3 extracted by ultrasonic–assisted (UA-GPP3)	892.7 kDa	Man:Rha:Glu:Gal:Xyl:Ara = 8.32 ± 0.09:9.01 ± 0.02:59.28 ± 0.11:4.33 ± 0.03:12.19 ± 0.12:6.87 ± 0.05	[46]
Rhizome	A neutral ginger polysaccharide fraction (NGP)	6.305 kDa	Glu:Gal:Ara = 93.88:3.27:1.67	[47]
Rhizome	A water extracted polysaccharides (WEP) containing fraction from ginger	36 kDa	N/A	[48]
Rhizome	Ginger polysaccharides were extracted by hot water extraction (HGP)	1831.75 kDa	Man:Gal = 3.1:0.9	[43]
Rhizome	Ginger polysaccharides were extracted by enzyme assisted extraction (EGP1)	11.81 kDa	Man:Glu:Gal:Ara = 13.3:80.7:4.0:2.0	[43]
Rhizome	Ginger polysaccharides were extracted by enzyme assisted extraction (EGP2)	688.73 kDa	Man:Rha:Glu:Gal:Xyl:Ara = 49.4:0.8:32.6:7.7:2.5:7.0	[43]
Rhizome	Ginger polysaccharides were extracted by ultrasonic cell grinder extraction (UGP1)	769.19 kDa	Man:Glu:Gal:Ara = 28.0:59.2:9.6:3.2	[43]
Rhizome	Ginger polysaccharides were extracted by ultrasonic cell grinder extraction (UGP2)	1432.80 kDa	Man:Rha:Glu:Gal:Xyl:Ara = 27.2:2.2:12.0:26.3:10.5:21.7	[43]
Stems and leaves	Polysaccharides from ginger stems and leaves (GSLP) by hot water extraction (HWE-GSLP)	N/A	Man:Rha:Glu:Gal:Xyl:Ara:GlcA:GalA = 1.95:17.22:4.69:38.88:5.66:28.42:1.81:1.34	[32]
Stems and leaves	Polysaccharides from ginger stems and leaves (GSLP) by ultrasound-assisted extraction (UAE-GSLP)	N/A	Man:Rha:Glc:Gal:Xyl:Ara:GlcA:GalA = 2.11:16.06:7.518:32.44:7.764:28.43:1.74:3.95	[32]
Stems and leaves	Polysaccharides from ginger stems and leaves (GSLP) by alkaline solution extraction (ASE-GSLP)	N/A	Man:Rha:Glc:Gal:Xyl:Ara:GlcA:GalA = 2.20:16.24:7.453:34.09:6.36:26.72:1.98:4.96	[32]
Stems and leaves	Polysaccharides from ginger stems and leaves (GSLP) by enzyme-assisted extraction (EAE-GSLP)	N/A	Man:Rha:Glc:Gal:Xyl:Ara:GlcA:GalA = 1.47:9.63:3.84:15.31:27.94:35.55:3.68:2.586	[32]
Rhizome	A novel polysaccharide (ZOP) was extracted from Zingiber officinale	6040 kDa (7.17 %) and 5.42 kDa (92.83 %)	GlcA:GalA:Glu:Gal:Ara = 1.97:1.15:94.33:1.48:1.07	[49]
Rhizome	Zingiber officinale polysaccharides (ZOP)	6040 kDa (7.17 %) and 5.42 kDa (92.83 %)	GlcA:GalA:Glc:Gal:Ara = 1.97:1.15:94.33:1.48:1.07	[49]
Rhizome	Zingiber officinale polysaccharides 1 (ZOP-1)	837 kDa	Glc:Gal:Ara = 1.00:95.09:2.26	[49]
Rhizome	Ginger polysaccharides (GP)	747.2 kDa	Man:Rha:GlcA:GalA:Glc:Gal:Xyl:Ara:Fuc = 0.17:0.13:0.21:0.12:0.08:1:0.16:0.64:0.18	[29]
Rhizome	Ginger polysaccharides UGP1	1002 kDa	N/A	[54]
Rhizome	Ginger polysaccharides UGP2	1296 kDa	N/A	[54]

N/A means not mentioned.

## 4. Biological Activities of Ginger Polysaccharides

Many studies, including in vivo and in vitro experiments, have confirmed that ginger polysaccharides have rich biological activities, including anti-influenza, anti-colitis, anti-tussive, anti-oxidant, and anti-tumor effects. The specific biological activities of ginger polysaccharides are summarized in Table 3.

### 4.1. Anti-Oxidant Effects

Oxygen free radicals have been proved to be associated with Alzheimer’s disease, atherosclerosis, diabetes, radiation damage, and other diseases. If anti-oxidants are carried out early, the occurrence of such diseases can be prevented [55,56]. Scientific research has proved that ginger polysaccharide has an anti-oxidant effect. In a study on the anti-oxidant effects of ginger polysaccharide ZOP and ZOP-1, the anti-oxidant effects of ZOP and ZOP-1 were evaluated in vitro by measuring the scavenging ability of DPPH• and OH•, chelating ability of Fe^2+^, and total reducing ability of ZOP and ZOP-1 with VC as positive control. The results showed that the scavenging activity of ginger polysaccharide on OH• and DPPH• increased with the increase of its concentration. When ZOP, ZOP-1, and VC were at the same concentration (1600 μg/mL), the median maximum inhibitory concentration (IC_50_) for DPPH was 226.8, 93.6, and 37.0 μg/mL, respectively. When ZOP and ZOP-1 were 1600 μg/mL, the scavenging ability of OH• was 33.6% and 99.1%, respectively. When the chelating ability of ZOP and ZOP-1 to Fe^2+^ was measured, the activity of ZOP was 7.45 times that of ZOP-1 at the same concentration, and the difference was significant. The IC_50_ values of ZOP, ZOP-1, and VC were 0.149, 0.020. and 0.023 μg/mL, respectively. In addition, its total reduction capacity also increased with the increase of polysaccharide concentration. In short, high concentrations of ZOP and ZOP-1 have strong anti-oxidant capacity [49]. Another study analyzed the anti-oxidant activity of ginger polysaccharides by measuring ABTS radical scavenging activity, DPPH radical scavenging activity, hydroxyl radical scavenging activity, superoxide radical scavenging activity, chelating activity, and reducing power assay. The results showed that the anti-oxidant capacity of HUE-GSLPs and UE-GSLPs polysaccharides was concentration dependent, and different preparation methods of polysaccharides could affect the anti-oxidant activity of ginger polysaccharide [38]. With the confirmation of the anti-oxidant activity of ginger polysaccharides from stems, people are also interested in the polysaccharides from ginger leaves. The anti-oxidant activity of ginger polysaccharides from stems and leaves (GSLP) was measured by free radical scavenging activity, including the anti-oxidation ability of ABTS, DPPH, hydroxyl, and superoxide free radicals, as well as the chelating activity and reduction ability of ferrous ions. By comparing the activity of ginger polysaccharides obtained by four different extraction methods, including hot water extraction (HWE), ultrasound-assisted extraction (UAE), alkaline solution extraction (ASE), and enzyme-assisted extraction (EAE), it was found that compared with other samples, the yield of ginger polysaccharides obtained by alkali extraction was the highest (11.38%), the molecular weight was the lowest, and the anti-oxidant activity was better [32]. The anti-oxidant activity of polysaccharides may be affected by various factors, such as molecular weight, monosaccharide composition, and glycoside linkage type. Therefore, this study shows that alkali solution extraction is a feasible method, which can extract polysaccharides from ginger stems and leaves with good anti-oxidant activity.

### 4.2. Anti-Tumor Effects

Ginger polysaccharide plays a vital role in controlling the proliferation and metastasis of tumor cells and promoting their apoptosis. In one study, the anti-tumor effect of ginger polysaccharide was evaluated by using MTT method. Three kinds of crude ginger polysaccharides (HCGP, ECGP, and UCGP) can be obtained from ginger rhizome after hot water extraction (HWE), enzyme assisted extraction (EAE) and ultrasonic cell grinder extraction (UCGE). After purification and separation, five purified polysaccharides were obtained, namely, HGP, EGP1, EGP2, UGP1 and UGP2. Studies have shown that the purified polysaccharide has a greater inhibitory effect on the proliferation of tumor cells than the corresponding crude polysaccharide. Among the five types of purified polysaccharides, UGP1 and UGP2 have a strong inhibitory effect on the proliferation of tumor cells. Among the five types of tumor cells (H1975, Hela, HCT116, B16, and MCF-7), UGP1 and UGP2 have significantly different inhibitory effects on their proliferation. UGP1 has a strong inhibitory effect on these kinds of tumor cells such as Hela and HCT116, of which the inhibitory effect of UGP1 on the human colon cancer HCT116 cell linen is relatively high (56.843 + 2.405%), which indicates that UGP1 may be a potential drug for the treatment of colon cancer [43].

### 4.3. Anti-Influenza Effects

Influenza is a respiratory disease caused by influenza virus and has strong infectivity and rapid transmission speed. Influenza does great harm to the human body, and influenza vaccination is an effective way to prevent disease [57,58]. Research shows that the mixed polysaccharides (MPs) extracted from shiitake mushroom, poriacocos, ginger, and tangerine peel are potential drugs to promote the immune response of influenza vaccine. In this study, a previously mouse-adapted A/WSN/33 (H1N1) influenza virus was used to establish the infected mouse model. The experiment was divided into three groups: control group, PBS group, and MPs group. There were 35 mice in each group, and they were intragastric treatment daily for a total of 30 days. On the 14th and 22nd days, the other two groups were subcutaneously injected with inactive influenza vaccine, except the control group. On the 30th day, 7 mice were sacrificed in each group, and the relevant parameters, including spleen index, lung index, spleen CD3^+^, spleen CD19^+^, and serum total IgG, IgG1, and IgG2a antibodies against A/WSN/33 (H1N1), were measured. Other mice were inoculated with influenza virus. After inoculation, seven mice were weighed and sacrificed on days 1, 3, and 5, respectively, and the relevant parameters, such as CD3^+^, CD19^+^, and CD278^+^ cells in lungs, lungs histology, virus titers of lungs, and serum INF-γ levels, were measured. The MPs pretreatment significantly increased the weight gain of the immunized mice after stimulation and reduced the clinical symptoms and lung injury of the immunized mice. These results showed that the MPs pretreatment could improve the anti-virus infection ability of immunized mice. Previous studies have shown that severe H1N1 infections may be related to IgG2 deficiency. After immunization, the levels of IgG and IgG2a in the serum of mice treated with MPs were higher, indicating that the MPs may promote vaccination mainly by up-regulating the level of IgG2a [59]. In addition, the proportion of CD19^+^ and CD278^+^ in the lung of mice treated with MPs increased, indicating that the MPs pretreatment promoted the ability of B cells and Th1 cells. In addition, the team also conducted a study on the effects of mixed polysaccharides on non-immune mice. After mixed polysaccharide pretreatment, non-immune mice experienced no significant trend of weight loss, clinical symptoms, lung index, and lung injury after experiencing viral attack, and related indexes increased [60]. It can be seen that ginger polysaccharide alone can play a certain pharmacological effect, and it can also play a good role in the human body together with other polysaccharides, which is worthy of further study.

### 4.4. Anti-Colitis Effects

Ulcerative colitis (UC) is a chronic inflammatory digestive tract disease which is related to the severity of inflammation and has the characteristics of high recurrence rate and difficult cure. At present, the commonly used therapeutic drugs have certain side effects on human body, so people gradually seek compounds from natural sources, including polysaccharides, to treat UC [61,62,63]. In one study, the therapeutic effect of ginger polysaccharide (GP) on UC was investigated in mice with dextran sulfate sodium (DSS)-induced colitis. Eight-week-old male mice were randomly divided into a control group, model group, 5-ASA group, and GP group. In addition to the control group, the mice in other groups were infected with UC by drinking 3% DSS drinking water freely, and the models were made for 10 consecutive days. The 5-ASA group and GP groups were gavaged with 5-ASA and GP on days 1, 3, 5, 7, and 9, respectively. The control and model groups were gavaged with normal saline. We observed and recorded the weight, diarrhea, blood in stool, and other physiological symptoms of the mice every day, and we evaluated the disease activity index (DAI). On the 10th day, the eyeball blood of mice was collected, the length of the colon and the weight index of the spleen were measured, and the organs were histopathological examined. Fecal samples of mice were collected for gut microbial analysis. The degree of colon tissue damage and the expression of pro-inflammatory factors were also detected.

The results showed that the mouse model of DSS-induced colitis had the characteristics of decreased colon length, weight loss, and broken colon epithelium. After treatment with GP, the colon shortening was inhibited, and the weight loss trend and DAI index were alleviated. The pathological results showed that the model group mice had serious mucosal injury and fibrosis-related mucosal and submucosal collagen deposition, while the GP group showed that the disease process of the mice was delayed, and the GP significantly improved the degree of inflammation and the scope of lesions of the colon. It had protective effects on the injury induced by DSS. Using ELISA to detect the expression level of pro-inflammatory factors, such as TNF-α, IL-6, IL-1β, IL-17A, and IFN-γ in tissues, it was found that the expression level of pro-inflammatory factors increased significantly in the model group, and the expression level of pro-inflammatory factors decreased significantly after GP treatment. This indicates that GP can inhibit the pathological inflammation of colitis by down-regulating the expression of pro-inflammatory factors. To detect the effect of DSS induction on the intestinal flora of mice and the effect of GP on the intestinal flora of mice, 16S rRNA gene amplification sequencing in the fecal matter was used for analysis. The results showed that the microenvironment of the intestinal bio-community in the model group mice was destroyed, and the abundance of bacterial colonies and the uniformity and diversity of bacterial species were reduced. GP group restored the imbalance and change of intestinal flora induced by DSS at different classification levels. In addition, GP can also protect the epithelial barrier and significantly reduce the apoptosis of colon epithelial cells, which plays an important role in the treatment of colitis. Based on these results, it can be seen that GP can effectively alleviate the symptoms of UC, regulate intestinal inflammation by inhibiting the level of pro-inflammatory factors, maintain the integrity of intestinal barrier, and regulate intestinal flora [29]. In addition, Zingiber officinale polysaccharides (ZOP) and ZOP-1 also had a significant anti-inflammatory effect on RAW264.7 cells cultured in vitro. ZOP and ZOP-1 showed no cytotoxicity to RAW264.7 and could promote its proliferation at the concentration range of 10–500 μg/mL. Meanwhile, it also significantly enhanced the expression of NO, TNF-α, IL-1β, and IL-6 and showed a concentration-dependent effect. This indicated that ZOP and ZOP-1 had a good anti-inflammatory effect on RAW264.7 [49]. In summary, these results will provide a new idea for the comprehensive development and utilization of ginger polysaccharide, and also provide innovative options for the development of functional foods or therapeutic drugs.

### 4.5. Anti-Tussive Effects

Cough is a common clinical symptom and a protective reflection of the body, which helps to clear the foreign material and secretions from the airway, thus facilitating the control of infection [64,65]. However, frequent and severe coughing will still have a certain impact on the body, which may lead to syncope, urinary incontinence, pneumothorax, and even rib fracture. Clinically, codeine phosphate tablets are often used to treat frequent and severe dry cough with good effect, but long-term use has drug dependence and side effects. For this reason, safe and low-toxic compounds from natural sources have attracted researchers’ attention, and polysaccharides are no exception. An experimental study in vivo showed that water-extracted polysaccharides (WEP) containing fraction from ginger rhizome has an anti-tussive effect, and the curative effect is basically the same as that of codeine phosphate. In this study, guinea pigs were stimulated with citric acid aerosol, and then treated with WEP, vehicle (water for injection), and positive control codeine phosphate, respectively. The cough number of guinea pigs were observed and recorded at 0, 30, 60, 120, and 300 min. The incidence of cough number was decreased from 6.2 ± 0.43 (means ± SEM) at 0 min (before extract administration) to 2.25 ± 0.23 (means ± SEM) at 30 min after WEP administration, while the cough number of perorally administered codeine phosphate (10 mg/kg body weight) decreased from 5.5 ± 0.67 to 2.83 ± 0.70 (mean ± SEM) at the same time interval. WEP significantly inhibited the cough reflex of guinea pigs, and 25 mg/kg body weight WEP showed the maximum cough-suppressing activity at 30 min. In addition, the responsiveness of airway smooth muscle in vivo showed that WEP could reduce the number of coughs but did not affect the specific airway resistance of animals [48]. The above results provided a scientific basis for the traditional use of ginger.

**Table 3:** Biological activities of ginger polysaccharides and their underlying mechanisms of actions.

Biological Activities	Compound Name	In Vitro or In Vivo	Indicated Concentrations and Animal Experiments/Test System	Action or Mechanism	Ref.
Anti-oxidant effects	Polysaccharides from ginger stems and leaves (HWE-GSLP, UAE-GSLP, ASE-GSLP and EAE-GSLP)	In vitro	ABTS radical scavenging activity, DPPH radical scavenging activity, hydroxyl radical scavenging activity, superoxide radical scavenging activity, chelating activity, and ferric reducing power.	ABTS radical scavenging activity: These samples’ anti-oxidant capacities of ABTS, although better than those of HWE-GSLP and UAE-GSLP, did not exceed those of Ascorbic acid (VC); DPPH radical scavenging activity: ASE-GSLP (IC_50_ = 0.492 mg/mL) < EAE-GSLP (IC_50_ = 0.975 mg/mL) < UAE-GSLP (IC_50_ = 2.877 mg/mL) < HWE-GSLP (IC_50_ = 3.583 mg/mL); Hydroxyl radical scavenging activity: ASE-GSLP exhibited higher hydroxyl radical scavenging activity than the other polysaccharides; Superoxide radical scavenging activity: The experimental IC_50_ values followed the trend ASE-GSLP < EAE-GSLP < UAE-GSLP < HWE-GSLP.	[32,38,49]
				Chelating activity: The strong Fe^2+^ chelating activity of EAE-GSLP and UAE-GSLP might be partially due to the high contents of–COOH and C-O groups in their structures; Ferric reducing power: In solutions of concentrations between 0.25 and 5.0 mg/mL, the reducing power of the EAE-GSLP was the greatest, followed by ASE-GSLP.	
Anti-tumor effects	Five purified ginger polysaccharides were obtained, namely HGP, EGP1, EGP2, UGP1 and UGP2	In vitro	The human colon cancer HCT 116 cell line, human cervical cancer Hela cell line, human lung adenocarcinoma H1975 cell line, human breast cancer MCF-7 cell line, mouse melanoma B16 cell line	UGP1 has a strong inhibitory effect on these kinds of tumor cells such as Hela and HCT116, of which the inhibitory effect of UGP1 on the human colon cancer HCT116 cell linen is relatively high (56.843 + 2.405%), which indicates that UGP1 may be a potential drug for the treatment of colon cancer.	[43]
Anti-influenza effects	The mixed polysaccharides (MPs) extracted from shiitake mushroom, poriacocos, gin-ger, and tangerine peel	In vivo	0.342 g/mL, four-week-old female BALB/c mice	Immune mice: The levels of IgG and IgG2a in the serum of mice treated with MPs were higher.	[59,60]
				Non-immune mice: The CD3^+^, CD19^+^ and CD25^+^ cell proportions were up-regulated in thymus under MPs pretreatment.	
Anti-colitis effects	Ginger polysaccharides (GP)	In vivo	SPF grade eight-week-old male C57BL/6 mice	GP alleviated UC symptoms by inhibiting pro-inflammatory cytokines levels to regulate intestinal inflammation, repairing the intestinal barrier, as indicated by occludin-1 and ZOP-1, and regulating gut microbiota.	[29]
Anti-tussive effects	A water extracted ginger polysaccharides (WEP)	In vivo	25 and 50 mg/kg body weight, thirty adult healthy male TRIK strain guinea pigs	WEP could reduce the number of coughs effort but did not affect the specific airway resistance of animals.	[48]

## 5. Structure–Activity Relationship

Polysaccharides have a complex structure. At present, most of the research on polysaccharide focuses on the primary structure, but the higher structure is relatively little. Therefore, the study on the structure–activity relationship of ginger polysaccharide is also very superficial. Based on the existing literature, the structure–activity relationship of ginger polysaccharide was sorted. On the one hand, it can reduce the duplication of research, and on the other hand, it can provide the direction for future research.

Many studies have shown that the molecular weight of polysaccharides is closely related to a variety of biological activities. Proper molecular weight is one of the necessary conditions for polysaccharides to display pharmacological activities. The molecular weight and anti-oxidant activity of polysaccharides from ginger pomace (HW-GPP1, HW-GPP2, HW-GPP3, and UA-GPP1, UA-GPP2, UA-GPP3) extracted by two different extraction methods were different. The average molecular weight of HW-GPP1, HW-GPP2, and HW-GPP3, and UA-GPP1, UA-GPP2, and UA-GPP3 were calculated as 89.2, 939.8, 1007.9 and 40.6, 868.1, and 892.7 kDa, respectively. In anti-oxidant experiments, UA-GPP3 possessed the strongest scavenging ability on DPPH radicals, and UA-GPP2 exhibited the strongest scavenging ability on hydroxyl and superoxide radicals. It is reported in the literature that polysaccharides with lower molecular weights have more significant biological activity than higher molecular weight polysaccharides. Because polysaccharides with lower molecular weight are easier to enter the organism and play a role. When UA-GPP2 is compared with HW-GPP2, HW-GPP3, and UA-GPP3 in scavenging DPPH radicals, it was consistent with the view reported in the literature. When UA-GPP2 is compared with HW-GPP1 and UA-GPP1 in scavenging hydroxyl and superoxide radicals, it is contrary to this view [46]. Therefore, the relationship between molecular weight and activity needs to be further studied.

It is well known that the monosaccharide composition of plant polysaccharides is an important factor affecting their biological activities. When the monosaccharide composition is different, the pharmacological effect is different. Using the same extraction method, two monosaccharide polysaccharides, GP1 and GP2, were extracted from the same raw material. The monosaccharide composition of GP1 is mannose, glucose, and galactose. GP2 is composed of arabinose, mannose, glucose, and galactose. GP1 might have anti-tumor activity, while GP2 displayed DPPH scavenging ability at concentration of 2 mg/mL, with a clearance rate of 67.2% [45]. When the monosaccharide composition is different, even if it has the same pharmacological effect, its strength is different. The study showed that the two ginger monosaccharides, ZOP and ZOP-1, had significant anti-oxidant effects, but the anti-oxidant activity of ZOP-1 was significantly higher than that of ZOP. When the concentration of ZOP and ZOP-1 is 1600 μg/mL, in the DPPH radical-scavenging assay, their maximum clearance rates were 72.7 and 95.2, respectively. In the hydroxyl radical assay, their maximum clearance rates were 33.6 and 99.1, respectively. The reason for this difference may be that the content of Gal in ZOP-1 is higher than that in ZOP. The composition and proportion of ZOP and ZOP-1 were GlcA:GalA:Glc:Gal:Ara in a molar ratio of 1.97:1.15:94.33:1.48:1.07 and Glc:Gal:Ara in a molar ratio of 1.00:95.09:2.26, respectively. This speculation is consistent with the results of the anti-oxidant experiment of *Laminaria japonica* Aresch polysaccharide [49]. When the composition of monosaccharide is the same, the different molar ratio will also affect the biological activity of polysaccharide. ASE-GSLP and HWE-GSLP in ginger polysaccharide are composed of Man, Rha, Glc, Gal, Xyl, Ara, GlcA, and GalA, with the proportions of 2.20:16.24:7.45:34.09:6.36:26.72:1.98:4.96 and 1.95:17.22:4.69:38.88:5.66:28.42:1.81:1.34, respectively, which have different anti-oxidant activities. In the DPPH radical-scavenging assay, the experimental scavenging rate of 50% (IC_50_) values of ASE-GSLP are 0.492 mg/mL, and the IC_50_ of HWE-GSLP is 3.583 mg/mL. In the superoxide radical assay, the IC_50_ values followed the trend ASE-GSLP < HWE-GSLP [32].

Moreover, it has been proved that the main chain and conformation of ginger polysaccharide may also affect its biological activity. Ginger polysaccharide NGP, which had the main chain of 1,4-linked α-d-Glcp and α-d-Glcp residues branched at C-6 position, exhibited immunomodulatory activity [47]. The main chain of ginger polysaccharides HGP, EGP1, EGP2, UGP1 and UGP2 is →4)-α-d-Glc (1→ and -α-Man*p*-(1→, which has strong anti-tumor activity. Among them, EGP2 and UGP1 have special structure →6)-β-d-galactose-(1→β-d-galactopyranose, which has an anti-tumor effect, especially on human colon cancer cells [43]. In the structure–activity relationship, the conformation of ginger polysaccharide also needs attention. Congo red experiment showed that GP1 existed triple helical structure but GP2 existed random coils. GP1 has an anti-tumor effect, while GP2 has a scavenging effect on DPPH [45]. Not all natural polysaccharides have ideal biological activities. Chemical modification of polysaccharides can improve their pharmacological activities and help to study the structure–activity relationship of polysaccharides. Therefore, structural modification of polysaccharides has become a research hotspot. By modifying the structure of ginger polysaccharide, the biological activity of ginger polysaccharide can be enhanced or given new biological activity, and the application range of polysaccharide can be widened.

To sum up, a comprehensive understanding of the structure–activity relationship of ginger polysaccharides will help to better develop food supplements and clinical drugs based on ginger polysaccharides. Therefore, a lot of scientific research is urgently needed to solve this problem.

## 6. Applications of Ginger Polysaccharides

**Figure 3:**
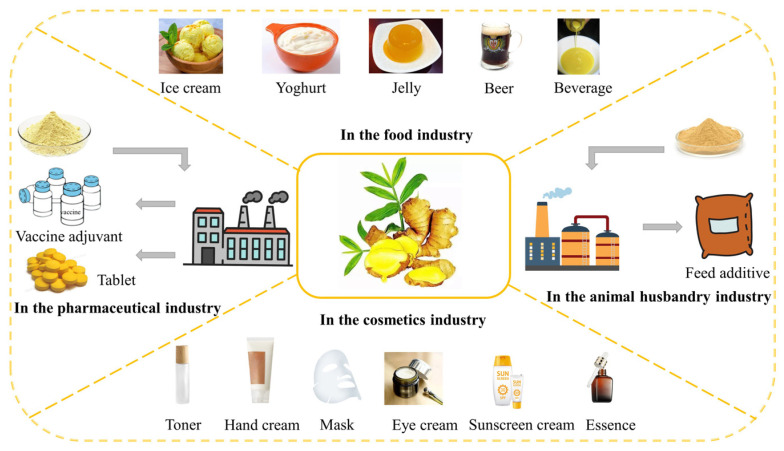
Practical applications of ginger polysaccharide.

Ginger polysaccharides can be used in a variety of fields, such as food, pharmaceuticals, cosmetics, and animal husbandry industry, to increase its nutritional and medicinal value after a series of processing processes (Figure 3).

### 6.1. In the Food Industry

With the development of the economy, the improvement of living conditions and the change of lifestyle, the consumption demand and purchasing trend of consumers have gradually changed. While purchasing food to eliminate hunger, consumers also hope that it can promote human health. Ginger, a medicinal and edible homologous plant, has a wide range of applications and is widely used in daily diet [66,67]. Among them, ginger polysaccharide, an important active ingredient, has certain research and development significance because of its high nutritional value, extensive pharmacological activities, and small side effects. Adding the proper amount of ginger polysaccharides to flour can improve the toughness, viscosity, and the color of the product, which can be used to produce bread, biscuits, noodles, and other products. In the preparation of ice cream, yoghurt and jelly, the addition of ginger polysaccharide can replace the emulsifier and stabilizer and can also improve the food flavor. Adding ginger polysaccharide to beer can maintain beer’s sensory characteristics on the one hand and enhance beer’s foam retention and health function on the other hand [68]. Ginger polysaccharide can also be used in the production of functional beverages. The addition of ginger polysaccharide will improve the taste, stability, and function of the beverage [69]. We must continue to strengthen the research on the activity of ginger polysaccharide so as to realize its large-scale and market-oriented application in the food field, meet the needs of different consumers, and give consumers sufficient choice space.

### 6.2. In the Pharmaceutical Industry

Since ancient times, natural medicine has been an important weapon for human beings to resist diseases. Plants are an important source of natural medicine. According to the long-term clinical practice and experience accumulation of traditional medicine in Chinese, Ayurveda, and Tibb-Unani, ginger is widely used to treat colds, fever, cough, sore throat, vomiting, carsickness, gastrointestinal complications, indigestion, constipation, arthritis, rheumatism, etc. In addition to traditional use, more and more scientific experiments have proved the anti-tumor effect of ginger in recent years. It can prevent and treat several types of cancer, including colorectal, prostate, breast, cervical, liver, and pancreatic cancer. The extensive pharmacological activity is closely related to its rich active ingredients, but the chemical composition of ginger is complex, and the pharmacological characteristics of each component are also different. Polysaccharide, as one of the important active ingredients, plays a vital role in the treatment of diseases [70,71]. Research shows that ginger polysaccharide can inhibit the growth of colon cancer by inhibiting the proliferation of colon cancer cells and inducing their apoptosis. It may be a potentially effective substance for the treatment and prevention of colon cancer. The development of natural medicine for the treatment and prevention of colon cancer has the advantages of high efficiency, small adverse reactions, a broad development prospect, and it is not easy to produce drug resistance.

Ginger can also be used in combination with other TCMs, for example, decoction of ginger and tangerine peel (*Citrus reticulata* Blanco) can be used to treat cough. Through water decoction, hydrophilic component polysaccharides from ginger are extracted, and modern pharmacological studies have shown that ginger polysaccharides have significant anti-tussive effects, which may be the key for this prescription to treat cough. It is worth mentioning that the use of ginger polysaccharides with other polysaccharides (Shiitake mushroom, poriacocos, and dried tangerine peel) also has certain pharmacological effects, and this mixed polysaccharide can serve as a potential influenza A (H1N1) vaccine adjuvant, promoting the body’s immune response, improving the efficacy of H1N1 vaccines, and enhancing the immune effect of vaccines [59,72,73]. It will also provide a new strategy by which to improve the effectiveness of the inactivated H1N1 vaccine against virus infection [74]. Ginger polysaccharides have been confirmed to have many benefits for human health in several studies. However, most of them are limited to applied research, and there are still gaps in the pharmaceutical industry of ginger polysaccharides, so ginger polysaccharides have an unlimited potential in medicine, have a large development space, and deserve deep research.

### 6.3. In the Cosmetics Industry

With the continuous improvement of people’s quality of life and the in-depth understanding of skin care knowledge, their requirements for cosmetics continue to develop to a higher level, and they are more focused on cosmetics from natural sources, i.e., raw materials, so TCM cosmetics are more popular [75,76,77]. Ginger polysaccharide has high safety and rich biological activity, which basically meets the needs of cosmetics consumers. Cleansing products such as facial cleanser, shower gel, and soap made from ginger polysaccharide can remove oil, clean pores, effectively relieve skin inflammation, and remove acne. Using ginger polysaccharide as a raw material to make eye cream, essence, sunscreen cream, facial mask, and other skin care products can play the role of whitening, sunscreen, anti-aging, and freckle removal. Because ginger polysaccharide has significant anti-oxidant activity, it can eliminate free radicals, such as DPPH and superoxide anion, and inhibit tyrosinase activity. Tyrosinase activity affects the formation of melanin, and melanin content plays a decisive role in the change of skin color. Ginger polysaccharides improve skin color by removing free radicals and reducing the amount of melanin. It can also prevent skin cells from being over-oxidized by removing free radicals, thus protecting skin cells and delaying skin aging. Ginger polysaccharide linked with a large number of hydrophilic hydroxyl groups can combine with water molecules in the form of hydrogen bonds, and they have a good film-forming property, which can reduce the evaporation of water on the skin surface. It can be developed into facial cream, hand cream, moisturizing cream, body cream, and toner. This kind of product has the dual effects of nourishing and moisturizing skin, and it will have a broad application prospect in cosmetics. With the expansion of the scope of TCM research, more and more natural products will be discovered and applied in the field of cosmetics. This will diversify cosmetics products and can meet the needs of different consumers.

### 6.4. In the Animal Husbandry Industry

Feed is the major component in the animal husbandry industry. Adding additives to feed can improve nutrient utilization, health index, and animal performance [78]. Feed additives such as antibiotics, preservatives, and hormones are effective, but they also have side effects [79,80,81]. For example, overuse of antibiotics as feed additives will cause drug residues in livestock, drug resistance, damage to the animal body, and environmental pollution. Ginger, as a feed additive, has the characteristics of high nutritional value, mild adverse effects, low cost, and no drug residues, which can effectively reduce the use of antibiotics and other drug additives, but the spicy taste limits its use. The polysaccharide extracted from ginger can be developed as a feed additive. On the one hand, it can reduce the stimulation of spicy taste. On the other hand, it can not only improve the immunity of the body, but also promote the appetite of animals, maintain the balance of intestinal flora, and promote the growth and development of animals. Therefore, ginger polysaccharide is an ideal feed additive with great potential. It plays a very important role in supplementing animal nutrition and animal medical treatment. Ginger polysaccharide use is of great significance to promote the safety and green development of animal husbandry.

## 7. Conclusions and Perspectives

**Figure 4:**
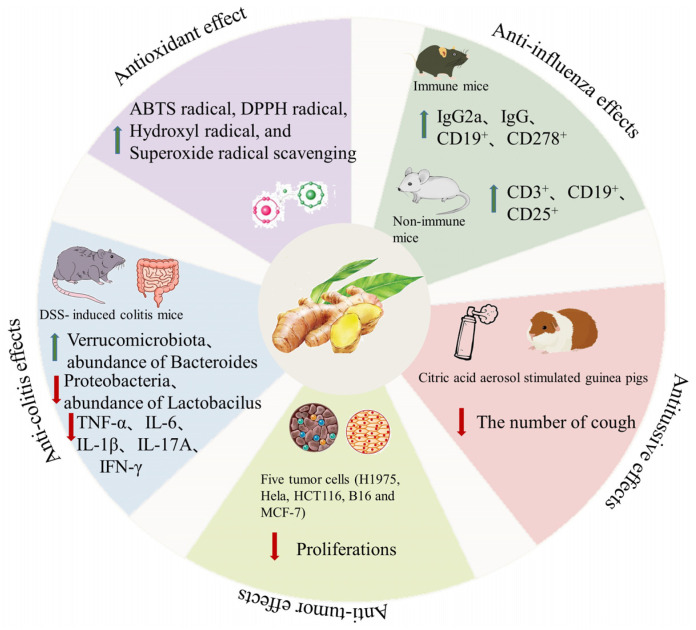
Biological activities of polysaccharides from ginger. (The red arrow indicates a downward revision. The green arrow indicates an upward revision).

Ginger is rich in nutrition. Its unique taste and fragrant smell enrich the taste of other vegetables, and it is loved by people and becomes an important seasoning for cooking. Ginger is not only rich in nutrients, but also an important source of natural high-quality polysaccharide. At present, there are many methods for ginger polysaccharide extraction, each of which has its own characteristics. The main methods are solvent extraction, enzyme-assisted extraction, microwave-assisted extraction, and ultrasonic-assisted extraction. With the development of science and technology and the integration of multi-disciplines, the extraction methods for obtaining a higher extraction rate and ensuring that the biological activity of polysaccharides is not destroyed as much as possible are constantly being explored and optimized. In addition, toxic or carcinogenic reagents are often used in the current extraction methods. Whether a safer and green purification and separation method can be found is a problem to be solved. The structure of ginger polysaccharides is complex. The current research shows that the structure of ginger polysaccharides is RGI type, but other research results show that there are other structures; there is still a lot of room for the research into the structure of ginger polysaccharides. In recent years, ginger polysaccharide has attracted extensive attention in the biological and medical fields because of its rich biological characteristics and pharmacological effects. It has shown good biological activities in vivo and in vitro, including anti-influenza, anti-colitis, anti-tussive, anti-oxidant, and anti-tumor effects. The detailed pharmacological action is shown in Figure 4. Among them, ginger polysaccharide plays a very important role in inhibiting colon inflammation, regulating intestinal microorganisms, and inhibiting colon cancer cell proliferation, and the specific mechanism of action is worthy of further study. Although the research on ginger polysaccharide has been uninterrupted, the research on its structure and biological activity still needs to be further explored. In the future, the structure of ginger polysaccharide can be modified to improve its inherent biological activity or produce new biological activity. Moreover, it is crucial to strengthen the research on the structure–activity relationship of the modified ginger polysaccharide and to clarify the correlation between the structural characterization of the modified site, quantity, and activity.

Ginger polysaccharide has been explored and developed in the food, cosmetics, animal husbandry industries in addition to being a concern in the field of medicine because of its various biological activities. This provides a broad market for the development and utilization of ginger polysaccharides and provides a good opportunity for the in-depth development of the rich ginger resources in countries that mainly produce ginger, such as India and China. However, opportunities and challenges coexist. The application of ginger polysaccharide in the cosmetics field requires more consideration of its safety, stability, and compliance with the quality control standards of cosmetics raw materials. In the field of food, we can consider developing products with different health functions, which are suitable for different groups, to make ginger polysaccharide-functional food personalized and diversified. In the field of medicine, the research trend of ginger polysaccharide can consider the evaluation of the vaccine adjuvant effect, the in-depth study of its adjuvant mechanism, the study of the possibility and mechanism of compatibility with respect to other adjuvants, and the study of its structure and structure–activity relationship. This in-depth and systematic study of ginger polysaccharides can help people understand and apply ginger polysaccharides from more perspectives.

Although ginger polysaccharide resources have been gradually developed and utilized, most of them are developed and utilized separately for the rhizome; thus, we lack the comprehensive and centralized utilization of ginger after its harvest and processing. Previous studies have shown that ginger leaves and ginger dregs also contain rich active ingredients which have a certain medicinal value and development potential. By analyzing the composition and function of ginger by-products, as well as their application space, the resultant secondary and multiple utilizations of ginger by-products can form a series and large-scale product developments and utilization approaches, which can not only achieve an efficient use of resources but also protect the environment and improve the economic benefits of enterprises.

At this stage, the extraction and purification technology of ginger polysaccharide has gradually improved, and the type and structure of ginger polysaccharide has been gradually identified. In the future, the mechanism of its action in the body will be further studied so as to better apply it to functional foods, drugs, and cosmetics, and expand it to the animal husbandry industry, realizing the full use of ginger polysaccharide, while providing reference and direction for the development of other polysaccharides.

## Data Availability

Not applicable.
